# A morphing approach for continuous generalization of linear map features

**DOI:** 10.1371/journal.pone.0243328

**Published:** 2020-12-08

**Authors:** Aji Gao, Jingzhong Li, Kai Chen

**Affiliations:** 1 School of Resource and Environment Sciences, Wuhan University, Wuhan, China; 2 Chengdu Research Institute of Surveying and Investigation, Chengdu Sichuan, China; Universidade Federal de Uberlandia, BRAZIL

## Abstract

With the development of web maps, people are no longer satisfied with fixed and limited scale map services but want to obtain personalized and arbitrary scale map data. Continuous map generalization technology can be used to generate arbitrary scale map data. This paper proposes a morphing method for continuously generalizing linear map features using shape context matching and hierarchical interpolation (SCM-HI). More specifically, shape characteristics are quantitatively described by shape context on which shape similarity is measured based on a chi-square method; then, two levels of interpolation, skeleton and detail interpolations, are employed to generate the geometry of intermediate curves. The main contributions of our approach include (1) exploiting both the geometry and spatial structure of a vector curve in shape matching by using shape context, and (2) preserving both the main shape structure as-rigid-as-possible and local geometric details as gradual and smooth as possible for intermediate curves by hierarchical interpolation. Experiments show that our method generates plausible morphing effects and can thus serve as a robust approach for continuous generalization of linear map features.

## Introduction

With the development of the internet, maps are widely used on desktop computers, web pages and mobile terminals. Internet map users are very diverse, and different users have different requirements for map scales. Traditional multiscale map databases have difficulty meeting the manifold needs of map users. As such, continuous map generalization, which can generate maps at any scale, has become an important research topic in the field of cartography and geographical information science.

One solution for continuous map generalization is a hierarchical data structure. There are some hierarchical data structures that support continuous generalization and multiscale representation, which include strip-tree [1], arc-tree [2], BLG-tree [3], GAP-tree [4, 5], tGAP-tree [6], the 3D SSC model [7] and the 5D model [8].

Another solution for continuous map generalization is adapted to map generalization algorithms. Van Kreveld [9] adopted several generalization operators (elimination, simplification, smoothing, enhancement, refinement, exaggeration, displacement, merging, aggregation, dissolution, collapse, typification, and classification) for continuous zooming. Cecconi and Galanda [10] proposed an adaptive zooming and continuous generalization model by combining hierarchical data structures and on-the-fly generalization, where the former approach is for objects requiring expensive computation and the latter for objects that can be generalized using simple methods and algorithms. Sester and Brenner [11] proposed a building continuous generalization method by decomposing traditional generalization operations (offset, extrusion, corner and typification) into simple operations (vertex insertion, deletion and removal) to display maps on mobile and small screen devices. Under the client-server framework, Yang, Ross and Weibel [12] proposed a model to constrain the removal (on the server side) and reconstruction (on the client side) of vertices, which can be used for progressive transmission and multiscale representation of vector data. Liu et al. [13] transformed the vector curves into Fourier series and then used a filtering method to continuously generalize cartographic curves for progressive transmission on the internet.

The bottlenecks of the relevant studies result from hierarchical data structures that cannot truly generate map data at arbitrary scales (they can only provide limited levels of details), and only a few of the traditional map generalization operators can be adapted for continuous generalization. Neither the hierarchical data structures nor the adapted generalization algorithms meet the requirements of on-demand mapping [14]. Therefore, we introduce a continuous map generalization solution called morphing technology.

Morphing is an important shape interpolation technique in the fields of computer graphics and computer vision [15]. It is a process of gradual and smooth transformation between a source and a target shape [16]. The effect of morphing transformation coincides with the idea of continuous map generalization [9, 14, 17–21]. Recently, researchers in the fields of cartography and geographical information science have used morphing techniques to construct continuous generalization models [11, 19, 20, 22–26]. The basic idea is to obtain a map between two key (or anchor) scales by morphing [19, 20, 27]. The process involves two main steps: characteristic correspondence and shape interpolation, where the structural characteristics of the source and target objects are matched through correspondence and the geometric coordinates of intermediate objects are generated by interpolation [22, 28, 29].

This study proposes an approach for continuous generalization of linear features using morphing. During the process of shape matching, both the geometry and spatial structure of each vertex on a shape are measured. An optimization technique is then used to seek the best matching. During the process of shape interpolation, two levels of interpolation algorithms are used to preserve both overall and local properties of intermediate shapes. The main novelty of our method lies in (1) an effective characteristics corresponding model based on shape context matching technique, and (2) a hierarchical interpolation model where both the skeletons and details are interpolated well. The rest of this paper is organized as follows. Section 2 reviews related research in morphing and its application in continuous map generalization. Section 3 details our model, which is based on shape context matching, and hierarchical interpolation (SCM-HI). Section 4 shows the experimental results and some conclusions are drawn in Section 5.

## Related works

Research on continuous generalization by morphing mainly focuses on two aspects: shape characteristics matching and trajectory interpolation.

The matching of shape characteristics can be treated as an optimization problem based on some similarity measurements. As an optimization problem, several optimization techniques have been used to seek an optimal correspondence. Sederberg and Greenwood developed a method to achieve shape correspondence by a dynamic programming technique [30]. Liu et al. (2004) [31] designed a metric to describe a shape property and then used a principal component analysis technique to seek the match between shapes. Based on the idea of template matching, Zhang (1996) proposed a fuzzy clustering method to achieve shape similarity measurement and matching [32]. Mortara and Spagnuolo (2001) extracted approximate skeletons of two anchor shapes and then established vertex correspondence by matching their skeletons [33]. Nöellenburg et al. (2008) used a dynamic programming technique to build global feature correspondence [22]. Wang et al. (2015) mapped a triangular mesh onto a rectangular regular array of an image, such that the reconstructed mesh produces no sampling errors [34]. Deng and Peng (2015) divided linear features into bends and developed a matching model based on bend similarity measurements [29]. Buchin et al. (2016) presented a territorial outlines simplification algorithm based on an operation called edge-move without changing area or topology [35]. Li, Ai, Liu and Yang (2017) proposed a simulated annealing-based model for morphing linear features [24]. Li, Li and Xie (2017) used a turning angle function to calculate building footprint similarity and obtain characteristics matching [25]. Generally, the above methods do not consider the map representation differences of spatial data at different scales. For the same geographic features on different map scales, their geometric shapes may be quite different because of generalization operations. It is therefore difficult to build characteristics correspondence for map features. To obtain a reasonable matching, not only the coordinates and orientation but also the context and local structural characteristics of a point should be considered.

For trajectory interpolation, considerable research has been performed to preserve the geometric and topological characteristics of intermediate shapes. To avoid the problem of shape shrinkage, Sederberg, Gao, Wang and Mu (1993) defined a polygon in terms of its edge length and the angles at its vertices, and then interpolated both edge lengths and point angles [36]. This method, however, only preserves the boundary and the interior of the input shapes may still be distorted. Then, as-rigid-as-possible [37, 38] and as-isometric-as-possible [37, 39] interpolation methods were proposed. The former uses rigid motion and compatible triangulation to improve the effect of interpolation, and the latter uses an isometry-invariant intrinsic coordinate system to simplify nonlinear isometric interpolation into a simple linear algebraic problem to achieve an effective isometric interpolation. These studies considered both the boundary and interior of input shapes during interpolation. Therefore, the problem of shape shrinkage was solved. Ma et al. (2018) proposed a method to deform a generalized cylinder based on its skeleton composed of a centerline and orthogonal cross sections, which avoided self-intersection [40]. Li et al. (2018) also proposed a morphing method based on Fourier transformation, where vector curves were first transformed into a Fourier series and then combined to obtain intermediate shapes [26]. Shen, Ai, and Li (2019) used a superpixel segmentation method to simplify urban buildings, which partially obtained continuous maps. The method does not need characteristics matching as it cannot distinguish between the main and auxiliary characteristics of a linear feature [41]. For geographical features, it is not enough to avoid shape shrinkage and self-intersection. The shape skeletons and geometric details of geographical features should be treated differently. Unfortunately, most existing morphing methods do not distinguish between the skeleton and details of interpolated shapes.

This paper proposes a morphing approach for continuous generalization of linear geographical features based on shape context matching and hierarchic interpolation, which builds reasonable characteristics correspondence and preserves shape features well.

## The SCM-HI model

**Fig 1:**
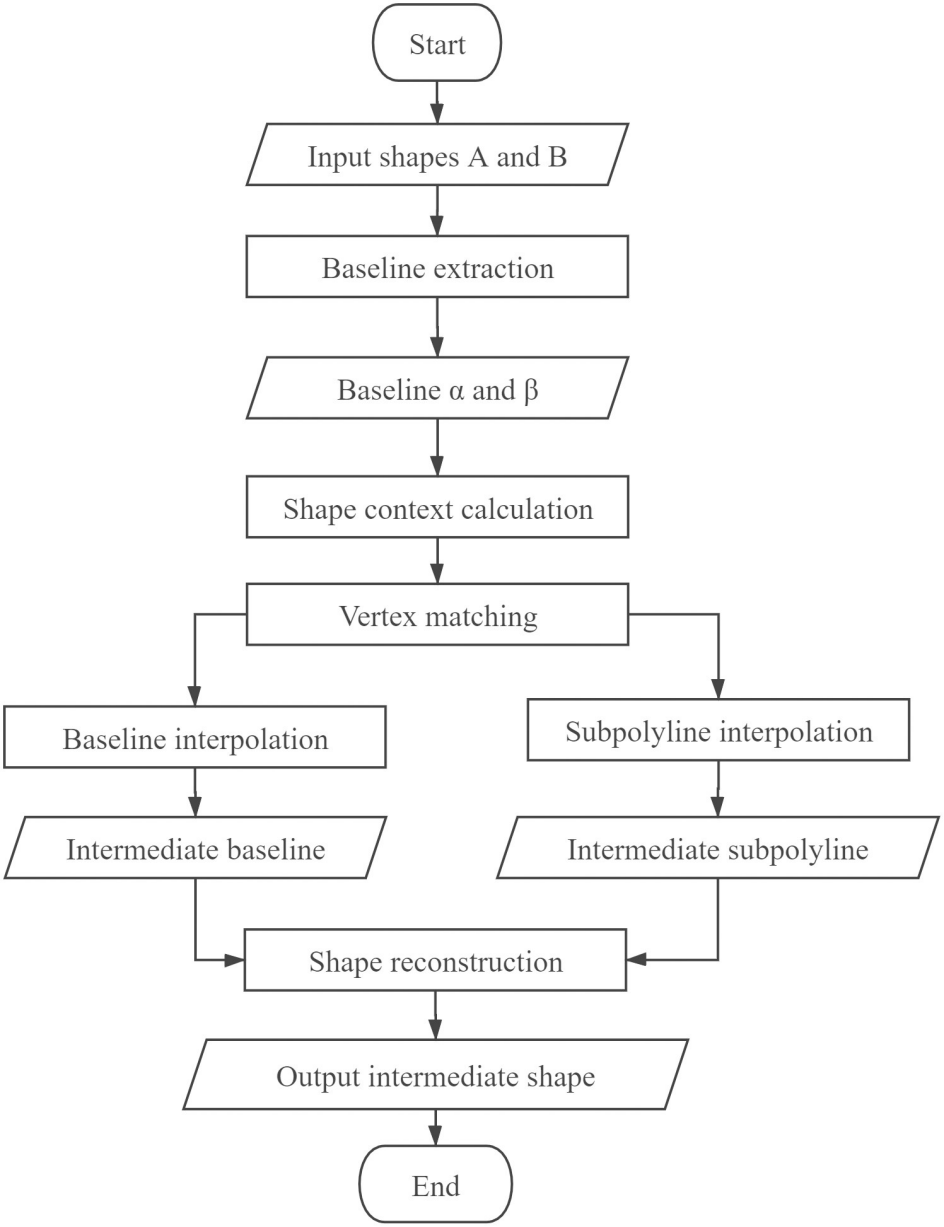
The flowchart of the SCM-HI model.

The proposed SCM-HI model includes six steps: baseline extraction, shape context calculation, vertex correspondence, baseline interpolation, subpolyline interpolation and shape reconstruction. The first step divides a curve into two different levels: baselines and subpolylines. A baseline is the main frame or skeleton of a curve, and the subpolylines are the geometry details between the vertices of the baseline. Each vertex on a baseline is measured by a shape context in the second step. An optimal baseline correspondence based on shape context is established by the Hungarian method [42] in the third step. After the correspondence, baselines and subpolylines are interpolated with different strategies in the fourth and fifth steps. Finally, an intermediate shape is reconstructed according to a gap-free principle in the final step. The specific algorithm process is shown in Fig 1.

### Baseline extraction

A baseline can be considered the skeleton or main frame of a linear feature. Baseline extraction serves two purposes: redundant point deletion and main frame preservation. We know that the sampling frequencies are different at different map scales. Generally, the larger the scale is, the higher the sampling frequency, and vice versa. During the matching process, redundant sampling points reduce the efficiency of matching algorithms and affect matching accuracy. During the interpolation process, while the topological and geometric characteristics of baselines should be preserved all the time, the geometric details of *subpolylines* can be changed smoothly. To improve matching efficiency, we need some data compression algorithms to obtain baselines by eliminating redundant data points.

**Fig 2:**
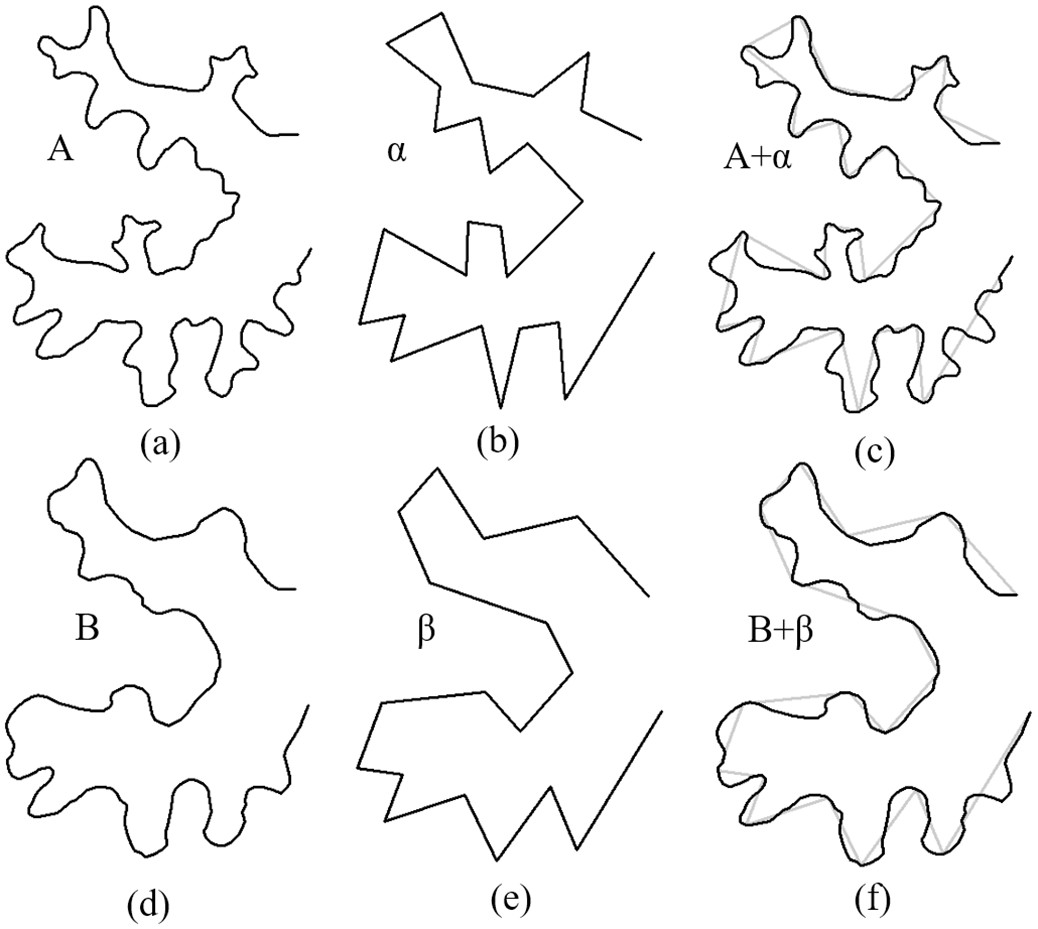
The process of baselines extraction. (a) Source curve A. (b) Baseline of the source curve A generated by the Douglas–Peucker algorithm. (c) Overlay of source curve A and its baseline. (d) Target curve B; (e) Baseline of the target curve B generated by the Douglas–Peucker algorithm; (f) Overlay of target curve B and its baseline.

In this paper, we use the Douglas–Peucker algorithm to obtain [43]. Given that the source and target curves are A (Fig 2(a)) and B (Fig 2(d)), and their corresponding baselines are *α* (Fig 2(b)) and *β* (Fig 2(e)) respectively, the vertices on *α* and *β* divide each original curve A and B into a series of subpolylines. Fig 2(c) and 2(f) show the overlay of the original curves (A and B) and their corresponding baselines (*α* and *β*). In the example, curve A is divided into 26 subpolylines, and curve B is divided into 18 subpolylines. The vertices of baseline *α* and *β* are used as the input vertices for shape context calculation and shape matching.

### Shape context calculation

Shape context, which has been widely used for object matching [44, 45], is a shape descriptor. The basic idea in shape context is that, when we characterize the shape property of a point on a curve, not only the point but also the spatial distribution of all its neighbor points are considered. Given a baseline *α*: {p_1_, p_2_, …, p_n_}, where *p*_*i*_ is the *i*^th^ point on the curve and *n* is the number of points on the curve, for any *p*_*i*_, its shape context can be obtained by the following steps. First, use *p*_*i*_ as the origin to construct a polar coordinate system where the angle *θ* is equally divided into S parts, and the logarithmic radius, i.e., log(*r*) is equally divided into T parts (Fig 2). The log-polar coordinate system is thus divided into S × T bins. Second, count the number of neighbor points located in each bin. For each bin *b*_*k*_, where *k* = 1, 2, …, S × T, its histogram *h*_*i*_(k) is calculated by the following formula:

$$\begin{array}{c} h_{i}\left(k\right)=\#\{p\neq p_{i}:p\in b_{k}\} \end{array}$$ (1)

Third, the series of *h*_*i*_(k)(*k* = 1, 2, …, *S* × T) is used to construct a vector c_*i*_ to represent the shape context of point *p*_*i*_.

$$\begin{array}{c} c_{i}=(h_{i}\left(1\right),h_{i}\left(2\right),\ldots ,h_{i}\left(S\times T\right)) \end{array}$$ (2)

Finally, the series of vectors *c*_*i*_(*i* = 1, 2, …, *n*) are used to construct an n × (S × T) matrix *C*_*α*_ as follows:

$$\begin{array}{c} \left[\begin{array}{cccc} h_{1}\left(1\right) & h_{1}\left(2\right) & \cdots & h_{1}\left(S\times T\right)\\ \vdots & \vdots & \vdots & \vdots \\ h_{i}\left(1\right) & h_{i}\left(2\right) & \cdots & h_{i}\left(S\times T\right)\\ \vdots & \vdots & \vdots & \vdots \\ h_{n}\left(1\right) & h_{n}\left(2\right) & \ldots & h_{n}\left(S\times T\right) \end{array}\right] \end{array}$$ (3)

To calculate shape context, there are four key parameters: S, T, Router, and Rinner where Router is the radius of the outermost circle, and Rinner is the radius of the innermost circle. According to Belongie et al. [44], the values of S and T are usually set to 12 and 5, respectively. The Router is typically set to 16(2^4^) times larger than the Rinner [46]. Given the maximum distance of all n*(*n* − 1)/2 point pairs on a curve of *n* points is *d*_max_ a we can set the *Router* = *d*_max_ + 1 Thus, all the vertices are included while calculating shape context.

**Fig 3:**
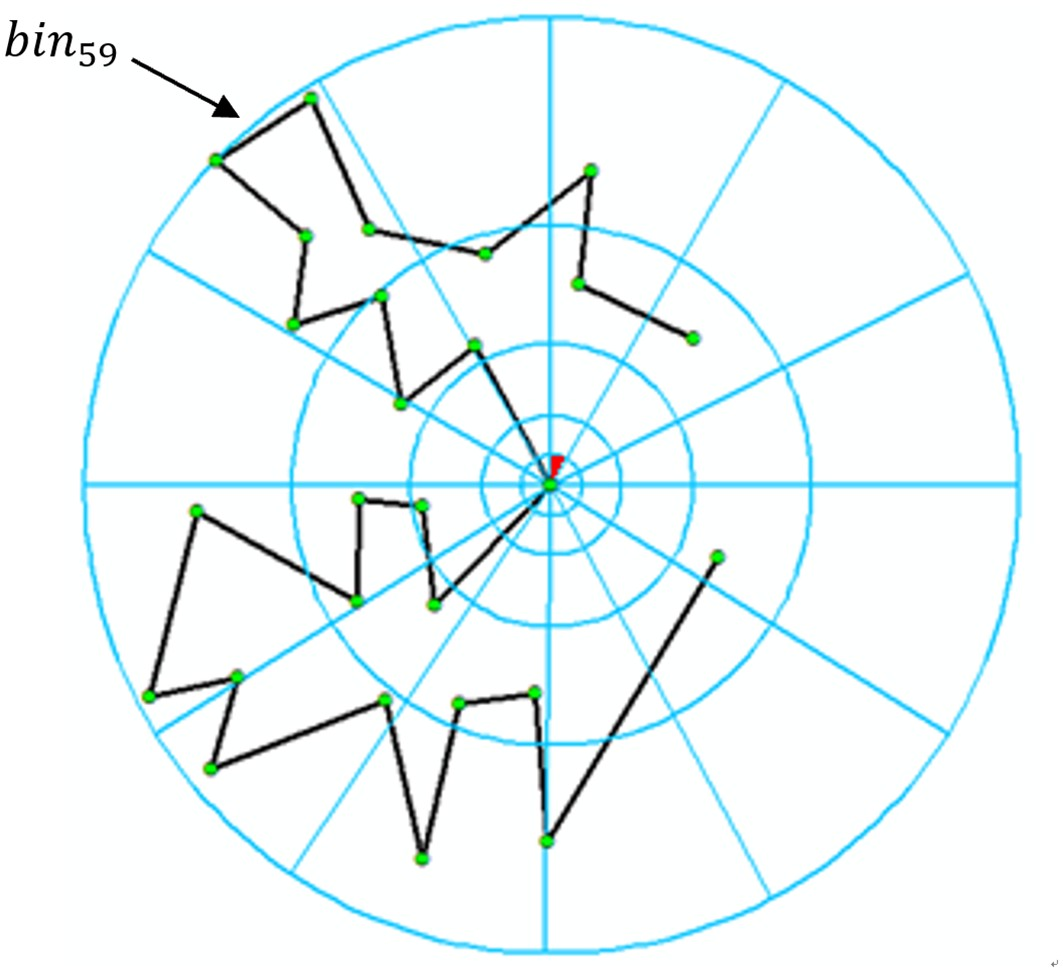
An example of calculating shape context.

Fig 3 illustrates the process of calculating shape context for the 15^th^ vertex on baseline *α*. There are a total of 60 bins (S = 12 and T = 5), which are numbered clockwise and from inside to outside. The red bin in the figure is the first bin. We calculate the value of *h*_15_(*k*) by counting the number of vertices inside the *k*^*th*^ bin, i.e., *h*_15_(59) = 5. After calculating *h*_15_(*k*)(1 ≤ *k* ≤ 60), we obtain vector *c*_15_, and after calculating *c*_*i*_(1 ≤ *i* ≤ 27), we obtain the matrix *C*_*α*_.

### Vertex matching

The distance between the shape contexts of two points on two separate baselines can be measured by the chi-squared statistic as follows [44]:

$$\begin{array}{c} c_{ij}=c(\alpha_{i},\beta_{j})=\frac{1}{2}\sum_{k=1}^{S\times T}\frac{{\left[h_{i}\left(k\right)-h_{j}\left(k\right)\right]}^{2}}{h_{i}\left(k\right)+h_{j}\left(k\right)} \end{array}$$ (4)

in which, *α*_*i*_ denotes the it point on baseline *α*, and *β*_*j*_ denotes the *j*^th^ point on baseline *β*. *h*_*i*_(k) and *h*_*j*_(k) denote the histogram of the k^th^ bin for the i^th^ point on baseline *α* and the *j*^th^ point on baseline *β*. Obviously, if the two points *α*_*i*_ and *β*_*j*_ are the corresponding points, the value of *c*_*ij*_ will be smaller than other pairs of points that do not correspond to each other.

Given a specific matching *π*, we can obtain the total matching cost between *α* and *β* by summing the *C*_ij_ between all pairs of corresponding *α*_*i*_ on *α* and *β*_*j*_ on *β*.

$$\begin{array}{c} H\left(\pi \right)=\sum_{i}C(\alpha_{i},\beta_{\pi (i)}) \end{array}$$ (5)

The consistent correspondence must be the one with the minimum total matching cost. The objective function of the matching can, therefore, be defined as:

$$\begin{array}{c} \min_{\pi }H\left(\pi \right) \end{array}$$ (6)

**Fig 4:**
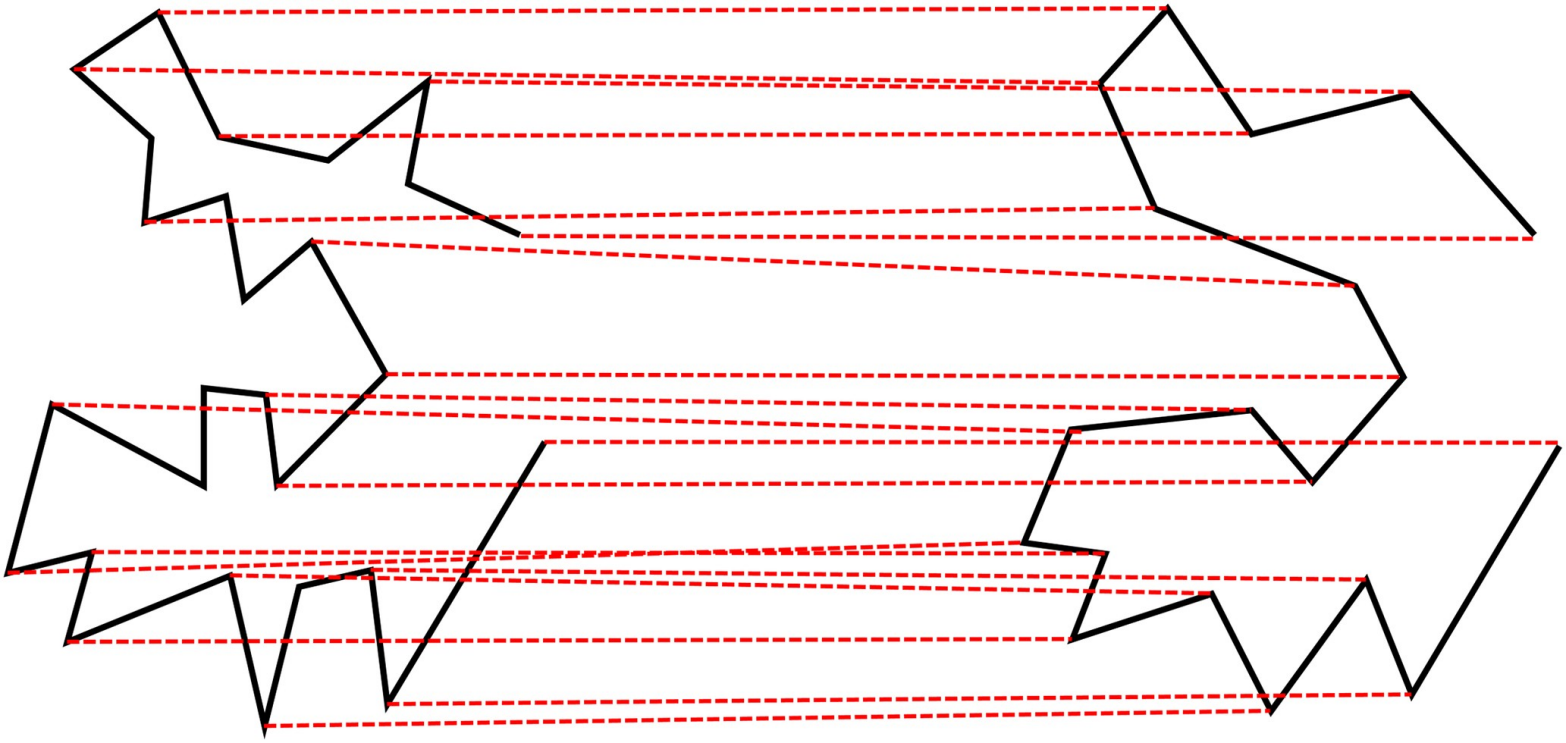
The best vertex matching between *α* and *β* The matched vertices are linked by red dashed lines.

Given that *N*_*α*_ and *N*_*β*_ are the numbers of points on baseline *α* and *β*, respectively. since *β* is a map generalization of *α*, *β* and has fewer details and therefore fewer points than *α* does, i.e., *N*_*α*_ ≥ *N*_*β*_, each point *β*_*i*_ on *β* can have a corresponding point *α*_*i*_ on *α*, but not vice versa. Therefore, the mapping from *β* to *α* becomes a bipartite graph G = (*α*, *β*, E), where every edge *e*_*ii*_ ∈ *E* has one endpoint in *α* and the other endpoint in *β*. Because *N*_*α*_ ≥ *N*_*β*_, our goal is to seek the optimal matching from *β* to *α* in the graph. In this study, we use the Hungarian method [42] for the best matching. Fig 4 shows the best matching found between the vertices of *α* and *β*, in which a red dashed line links a corresponding pair. Note that there are some vertices on *α* that have no matching vertex on *β*.

### Baseline interpolation

**Fig 5:**
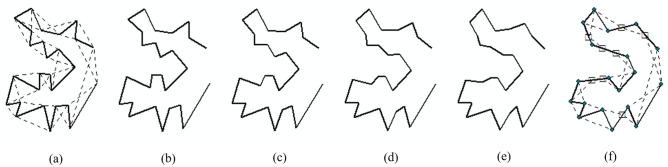
The interpolation of baselines. (a)*α*′ and its related triangles (dashed lines); (b)-(e) interpolated intermediate baselines; (f)*β*′ and its related triangles (dashed lines) where pseudovertices are marked as squares and real vertices are marked as circles.

To obtain an as-rigid-as-possible interpolation between two baselines *α* and *β*, not only the endpoints but also interior vertices should be considered. Some simplicial complexes can be used to cover both boundary and interior, such as the triangulation approach used by Alexa et al. [37] By connecting the first and the last vertex of *α* and *β*, we obtain two polygons *α*′ and *β*′, as shown in Fig 5(a) and 5(f). If we connect the end vertices of all three adjacent vertices on *α*′ and *β*′, we obtain a series of triangles, as shown in Fig 5(a) and 5(f). However, in most cases, *N*_*α*_ ≥ *N*_*β*_, so we need to add *N*_*α*_ − *N*_*β*_ pseudovertices to *β*′ based on the proportional chord-length principle [28]. In Fig 5(f), eight pseudovertices, which are marked as squares, are added to *β*′. Now that we have the same number of triangles for *α*′ and *β*′, a one-to-one correspondence can be made between two series of triangles of *α*′ and *β*′.

The next step is to obtain the least-distorted triangle-totriangle morphing between *α*′ and *β*′. We use the following constraints to construct the affine transformation between two corresponding triangles: the rotational angle should change linearly, and the resulting intermediate baseline should be simple [37]. In this approach, each vertex on *α*′ and *β*′ belongs to three different triangles. Each interpolation results in three different coordinates for a vertex. Here, we use the mean coordinate as the coordinate of each vertex on an intermediate baseline. Fig 5(b)–5(e) shows four intermediate baselines interpolated by this method.

### Subpolylines interpolation

Given a source subpolyline *S*_*i*_ of *m* vertices and its corresponding destination subpolyline *D*_*i*_ of n vertices, the vertices on both S_*i*_ and D_*i*_ can be normalized within [0, 1]. First, we calculate parameterized vertex sequences for $S_{i}=(t_{1}^{1},t_{2}^{1},\ldots ,t_{m}^{1})$ and $D_{i}=(t_{1}^{2},t_{2}^{2},\ldots ,t_{n}^{2})$ where $(t_{1}^{1}=t_{1}^{2}=0;t_{m}^{1}=t_{n}^{2}=1).$ Second, we obtain a new parameterized vertex sequence $T_{i}:({\bar{t}}_{1},{\bar{t}}_{2},\ldots ,{\bar{t}}_{M})\left(M<m+n\right)$ by merging *S*_*i*_ and D_*i*_ based on the proportional chord-length principle and only keeping the unique elements in the sequences. After merging, both *S*_*i*_ and *D*_*i*_ have *M* vertices and there is a one-to-one correspondence between their vertices.

To interpolate smooth and gradually changing subpolylines, a preferred method is to convert their Cartesian coordinates into an intrinsic parameter representation. According to Sederberg et al. [36], the intrinsic solution has the following advantages. First, it produces shape blends which generally are more satisfactory than those produced using linear or cubic curve paths. Second, it can avoid the shrinkage that normally occurs when rotating rigid bodies are linearly blended. Third, it can avoid kinks in the blend when there were none in the key polygons. Based on the idea of intrinsic solution, we used edge lengths and vertex angles to construct the intrinsic parameter sets for each pair of subpolylines. Given the intrinsic parameter sets of S_*i*_ and D_*i*_ are $f_{i}^{S}$ and $f_{i}^{D}$ respectively, based on Sederberg et al. [36], the intermediate subpolyline at scale *δ* can be interpolated based on the intrinsic parameter sets of $f_{i}^{S}$ and $f_{i}^{D}$

$$\begin{array}{c} f_{i}^{\delta }=\left(1-\delta \right)f_{i}^{S}+\delta f_{i}^{D} \end{array}$$ (7)

**Fig 6:**
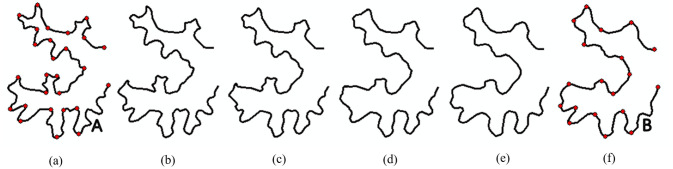
The interpolation of subpolylines. (a) Source curve A with 26 subpolylines; (b)–(e) Interpolated intermediate subpolylines at different scales. (f) Target curve B with 18 subpolylines.

Fig 6 shows an example of subpolylines interpolation. The source (Fig 6(a)) and target curves (Fig 6(f)) are divided into 26 and 18 subpolylines, respectively, based on the vertices on their baselines. They are then interpolated subpolyline by subpolyline.

### Shape reconstruction

**Fig 7:**
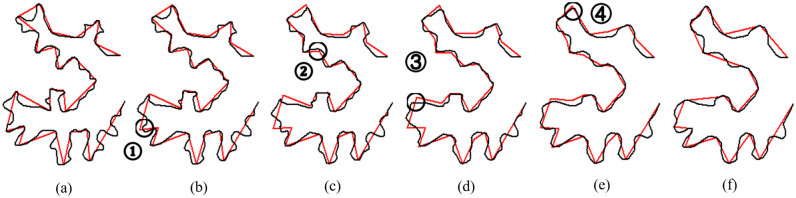
The overlaying of intermediate baselines and subpolylines. Example gaps between the endpoints of baselines and subpolylines are labeled as ① to ④.

**Fig 8:**
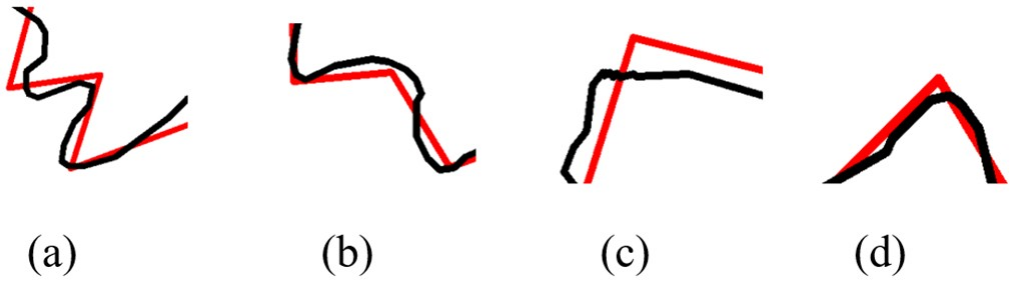
The close-up views of labeled gaps. Fig 8(a)–8(d) correspond to labeled gaps ① to ④ in Fig 7, respectively.

**Fig 9:**
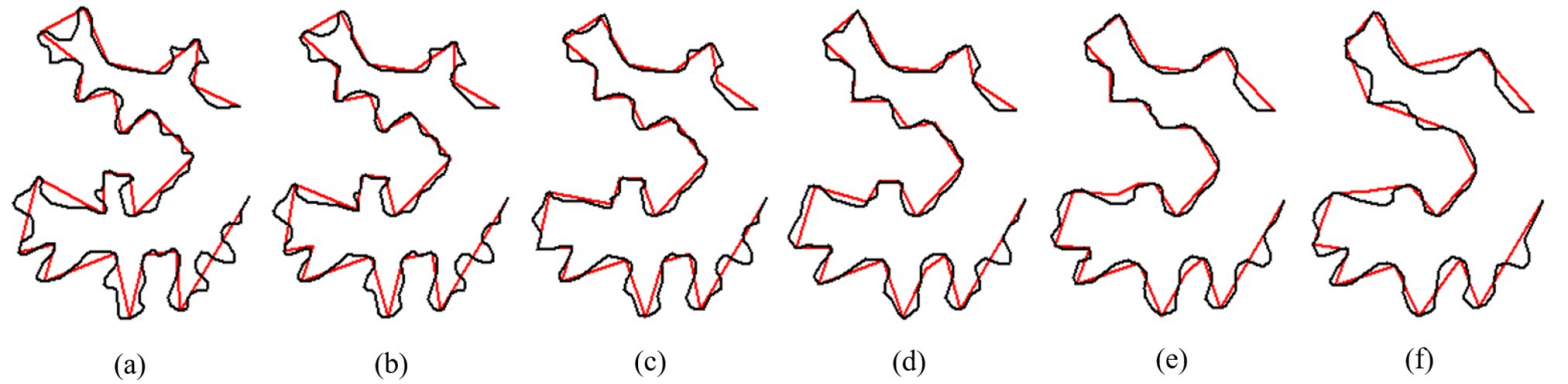
Interpolated curves after gap elimination and shape reconstruction.

When overlaying interpolated intermediate subpolylines with its baseline, we may see gaps at their endpoints. Example gaps, which are labeled as ① to ④, are shown in Fig 7. The gaps become more obvious in their close-up views in Fig 8. Those gaps are due to the inconsistency between their length and size. A simple and feasible solution is to adjust the length of a subpolyline based on the corresponding edge in the baseline and to align their endpoints to eliminate the gaps. After the adjustment, the intrinsic parameters of each intermediate subpolyline are converted back into the map coordinate system. Fig 9 shows the results of gap elimination and shape reconstruction. The main frame of input shapes is preserved well, and local geometry changes gradually as scale changes.

### SCM-HI based continuous generalization

For continuous map generalization, each intermediate curve has to be associated with a scale. Given that the scales of the source curve A, target curve B, and the intermediate curve Mid are *T*_*a*_, Tb and *T*_*b*_, respectively, a continuous map generalization can be denoted as [24]:

$$\begin{array}{c} Mid=f(A,B,g) \end{array}$$ (8)

where function f is the morphing model proposed in this paper, g is a parameter controlling the degree of interpolation. Mid is a curve between A and B, which is monotonic and continuous with respect to g which is 0 ≤ g ≤ 1. According to [24]:, the relationship between g and its map scale can be defined as follows:

$$\begin{array}{c} g=\frac{\frac{1}{T_{Mid}}-\frac{1}{T_{a}}}{\frac{1}{T_{b}}-\frac{1}{T_{a}}} \end{array}$$ (9)

where *T*_*Mid*_ is the intermediate scale and *T*_*a*_ and *T*_*b*_ are the anchor scales.

## Experiments and discussions

### Case studies

**Fig 10:**
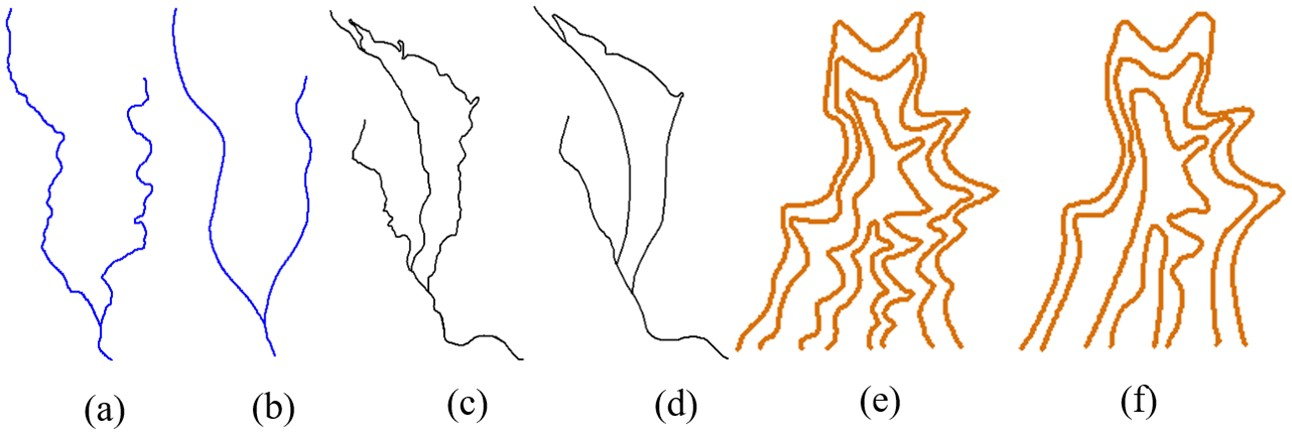
Three groups of experimental data. (a), (c), and (e) are the source shapes of river, road and contour lines, respectively, at the scale of 1:10,000. (b), (d), and (f) are the target shapes of river, road and contour lines, respectively, at the scale of 1:50,000.

The proposed model is tested for continuous generalization of linear map features. Three groups of typical linear map features, rivers, roads, and contour lines, are used in the experiment. These linear features are downloaded from National Fundamental Geographic Information System of China, and are generated from a fundamental dataset manually. Each group of datasets consists of one source shape and one target shape, and the scales of the source and target shapes are 1:10,000 and 1:50,000, respectively. The experimental datasets are shown in Fig 10.

**Fig 11:**
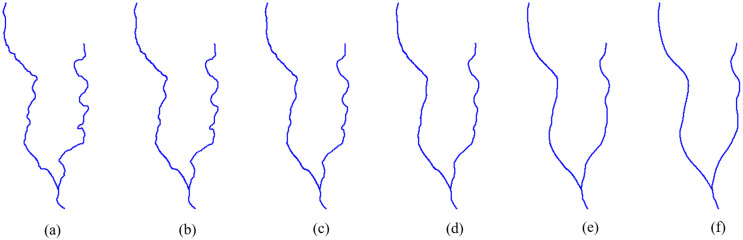
Continuous generalization of river features using the proposed method. The scales of the river features in (a) to (f) are 1:10,000, 1:18,000, 1:26,000, 1:34,000, 1:42,000, and 1:50,000, respectively.

**Fig 12:**
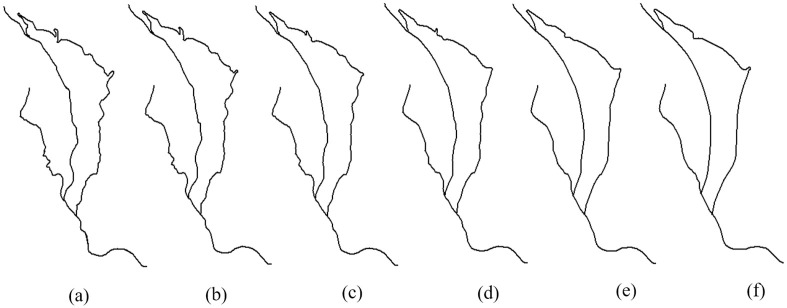
Continuous generalization of road features using the proposed method. The scales of road features in (a) to (f) are 1:10,000, 1:18,000, 1:26,000, 1:34,000, 1:42,000, and 1:50,000, respectively.

In the following experiments, the source and target scales T_a_ and T_b_ are 1:10,000 and 1:50,000, respectively. The sequence of intermediate scales T_Mid_ is 1:18,000, 1:26,000, 1:34,000, 1:42,000 and the corresponding g values are 0.2, 0.4, 0.6 and 0.8. The experimental results are shown in Figs 10–12.

**Fig 13:**
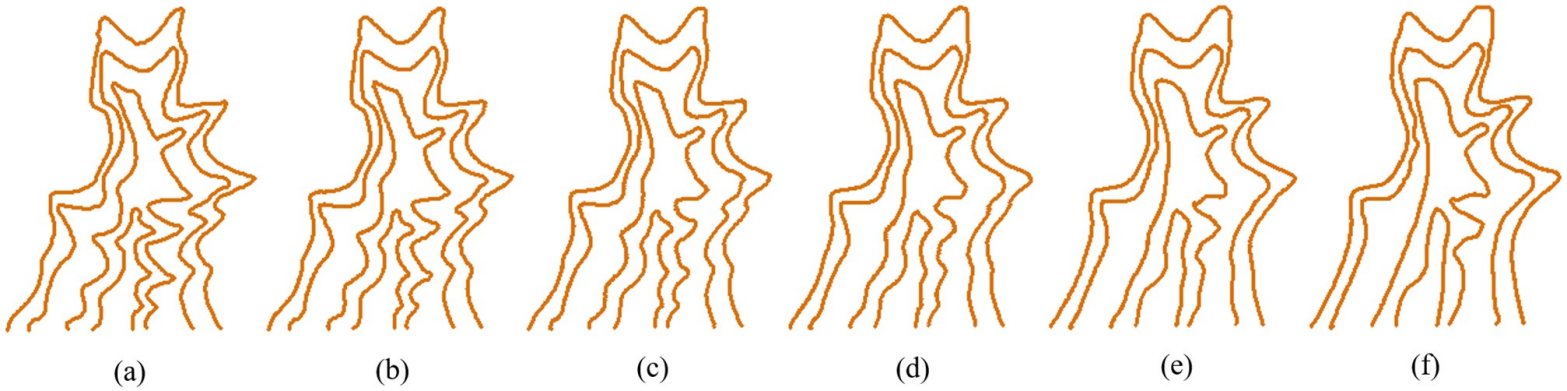
Continuous generalization of contour lines using the proposed method. The scales of contour lines in (a) to (f) are 1:10,000, 1:18,000, 1:26,000, 1:34,000, 1:42,000, and 1:50,000, respectively.

From the results, we can see that both topological structures and geometrical details are preserved well by our method. We know that groups of curved contour lines mean valleys or ridges, and they should have a nested geometric structure. As shown in Fig 13, all the contour lines maintain a reasonable gradient and their nested structure is maintained without any self-intersection. Since river and road features usually represent network structures, their connectivity is maintained in the generalization, as shown in Figs 11 and 12. Thus, due to better shape matching and hierarchical interpolation, not only are topological relationships well maintained, but the intermediate curves are also interpolated smoothly and continuously.

### Point accuracy analysis

The accuracy of the feature points of the linear feature after generalization can be quantitatively evaluated by C_tm1_ (Nöllenburg et al. [22]. Defining a function e: [0, 1] → E where e(u) = g(u) − f(u) and u ∈ [0, 1], the length |E| of the linear feature E is the value of C_tnl_ [29]. To evaluate the displacement of each point, we divide C_tn1_ by the number of coordinate points of the object to obtain a position accuracy value V_pa_. A smaller V_pa_ value indicates smaller point displacement.

We labeled three groups of experimental data: river, road and contour as G_1_, G_2_ and G_3_, respectively. We calculated the V_pa_ values between the intermediate shape and A (or B) on four scales (1:18,000, 1:26,000, 1:34,000 and 1:42,000) since the three groups of data have different numbers of objects, we calculate the V_pa_ values of each linear object separately and then calculate the average V_pa_ values of each group, which are recorded as *V*_pa1_ (intermediate shape and A) and *V*_pa2_ (intermediate shape and B). The experimental results are reported in Table 1. The unit of *V*_pa1_ and *V*_pa2_ is a meter.

**Table 1:** Statistics of position accuracy on different features at different scales.

	1:18,000		1:26,000		1:34,000		1:42,000	
	V_pa1_	V_pa2_	V_pa1_	V_pa2_	V_pa1_	V_pa2_	V_pa1_	V_pa2_
G_1_	**1.4**	2.6	1.8	2.2	2.1	1.9	2.5	1.5
G_2_	1.6	**3.5**	1.9	3.2	2.3	2.8	2.7	2.3
G_3_	1.5	3.1	1.9	2.8	2.2	2.3	2.6	1.8

From Table 1 we can see that the closer the intermediate scale is to 1:10,000, the smaller the value of *V*_pa1_, the larger the value of V_pa2_, and the closer the intermediate scale is to 1:50,000, the smaller the value of V_pa2_, the larger the value of V_pa1_. In the three groups of data, the V_pa_ value of the road feature is the largest, and that of the river feature is the smallest because the shape of the road feature is more complex, while the shape of the river feature is simpler. The minimum value of V_pa_ is V_pa1_ = 1.4, and the maximum value of V is V_pa_ is V_pa2_ = 3.5. The displacement error basically meets the requirement of the plane position accuracy of the related scale.

### Time complexity analysis

According to the section above, the SCM-HI model includes six steps. Therefore, the time cost of the SCM-HI model is also composed of six aspects. Two polylines are involved in the model. Assuming that the average number of vertices per polyline is N, then the time complexity of each step is O(NlogN), O(NlogN), O(N^2^), O(N), O(N) and O(log N). Therefore, our proposed algorithms have the complexity of O(NlogN) + O(NlogN) + O(N^2^) + O(N) + O(N) + O(log N). The algorithm of SCM-HI is implemented based on the platforms Microsoft Visual Studio (C#) 2019 and ArcGIS Engine 10.3, and performed on an Intel (R) Core(TM) i7-8550U @ 1.80GHz PC with 16 GB main memory running under Windows 10.

Table 2 shows the temporal statistics of the above experiment. The experimental data include 3 rivers, 6 roads and 4 contour lines. The average numbers of vertices of the linear features are 106 (river feature), 212 (road feature) and 151 (contour feature). For each group of experimental data, we repeat 100 times, and then use the average value as the time cost. T_1_, T_2_ and T_3_ correspond to the average time cost of river, road and contour experiments, respectively. In general, the SCM-HI model can generate approximately 10 intermediate polylines of moderate complexity per second.

**Table 2:** Statistics of time cost of SCM-HI model on different features.

	Step1	Step2	Step3	Step4	Step5	Step6	∑
T_1_	10	9	75	4	5	1	104
T_2_	11	12	85	5	8	1	122
T_3_	9	9	81	4	6	1	110

The runtime is measured in milliseconds.

### Application examples

**Fig 14:**
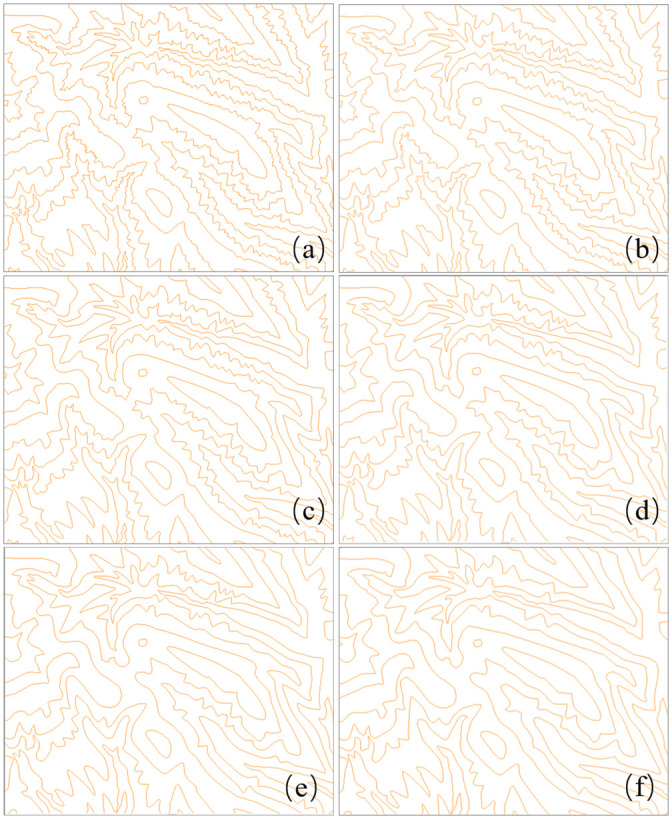
The application example of SCM-HI: Continuous generalization of a real contours map. The scales of the contours in (a) to (f) are 1:10,000, 1:18,000, 1:26,000, 1:34,000, 1:42,000, and 1:50,000, respectively.

After the above position accuracy and time complexity, the model is applied to real contour data for continuous generalization experiments. There are three reasons for choosing contour data. First, the shape characteristic of contours is more complex; second, the density of contours is higher; third, the geographical significance of contours is obvious, such as ridges and valleys expressed by group bends. Different from the traditional map generalization algorithm, we use structured interpolation to generate arbitrary intermediate scale data based on the data of one large scale and one small scale. The premise of this model is that the cardinality of large and small scales data must be equal. In this experiment, we delete the redundant data in the 1:10000 data compared with 1:50000, so that the object number in the two datasets is a one-to-one correspondence. Fig 14 shows the experimental results. The experimental results show that in the process of continuous generalization, the geometric bending morphology of contours is maintained well; the topological structure is not destroyed, and the geographical significance is preserved. There are 38 contours in the figure. The SCM-HI model is used to generate the data of four intermediate scales (1:18,000, 1:26,000, 1:34,000 and 1:42,000), and the cumulative time consumption is 3.3 seconds. Therefore, the SCM-HI model can be used for continuous generalization of real map data.

## Discussion

Theoretically, when the parameter g in formula (9) gradually changes from 0 to 1, the model can dynamically derive any intermediate scale data between the source and target scale. For example, given T_*a*_ = 1:10000 and T_*b*_ = 1:50000, when g = 0.375, we can get T_*mid*_ = 1:25000. When g = 0.5, we can get T_*mid*_ = 1:30000. Small change of g value will lead to small intermediate scale T_*mid*_ and intermediate shape Mid changes.

Technically, geometric and topological accuracy can be well maintained. Because any intermediate state is obtained by shape interpolation, its geometric accuracy is between that of the source and target objects. Moreover, by using intrinsic solution, the topology accuracy can be well maintained. This means that the SCM-HI model can avoid the shrinkage that normally occurs when rotating rigid bodies are linearly blended.

## Conclusions

This paper proposes a morphing method for continuously generalizing linear map features using shape context matching and hierarchical interpolation (SCM-HI). The SCM-HI model benefits from the context-based shape matching and two-level interpolation. The former uses shape context matching technology to control the structure of interpolation shape from geometry and spatial. The latter maintains both the main shape structure as-rigid-as-possible and local geometric details as gradual and smooth as possible for intermediate curves by hierarchical interpolation. It can be used for linear map features continuous generalization. The model can dynamically derive any intermediate scale data between the source and target scale and preserve the geometric and topological accuracy well.

Different from the traditional map generalization method, the SCM-HI model needs two shapes at a source and target scales as inputs. Not only that, to obtain good shapes at intermediate scales, features of the input shapes must be matched reasonably and interpolation must be carried out at different levels. In this paper, we use shape context and optimal matching to obtain vertex correspondence, and divide the interpolation process into two levels: as-rigid-as-possible interpolation for base lines and linear interpolation for sub-polylines.

The approach, however, has two limitations. First, it can only be used for continuous generalization of linear map features with both initial and target scales. Second, two sets of input data for interpolation must have the same cardinality. This means that if the source and target datasets have different numbers of map objects, then the number of objects generated by the model will be determined by the dataset with fewer objects. In the future, we plan to extend the method to other geometric dimensions.
